# Label-free liquid biopsy through the identification of tumor cells by machine learning-powered tomographic phase imaging flow cytometry

**DOI:** 10.1038/s41598-023-32110-9

**Published:** 2023-04-13

**Authors:** Daniele Pirone, Annalaura Montella, Daniele G. Sirico, Martina Mugnano, Massimiliano M. Villone, Vittorio Bianco, Lisa Miccio, Anna Maria Porcelli, Ivana Kurelac, Mario Capasso, Achille Iolascon, Pier Luca Maffettone, Pasquale Memmolo, Pietro Ferraro

**Affiliations:** 1 CNR-ISASI, Institute of Applied Sciences and Intelligent Systems “Eduardo Caianiello”, Via Campi Flegrei 34, 80078 Pozzuoli, Naples, Italy; 2 CEINGE Advanced Biotechnologies, Naples, Italy; 3 DMMBM, Department of Molecular Medicine and Medical Biotechnology, University of Naples “Federico II”, Naples, Italy; 4 Department of Chemical, Materials and Production Engineering, DICMaPI, University of Naples “Federico II”, Piazzale Tecchio 80, 80125 Naples, Italy; 5 Department of Pharmacy and Biotechnology (FABIT), University of Bologna, Bologna, Italy; 6 Interdepartmental Centre for Industrial Research ‘Scienze Della Vita e Tecnologie per La Salute’, University of Bologna, Bologna, Italy; 7 Centre for Applied Biomedical Research (CRBA), University of Bologna, Bologna, Italy; 8 DIMEC, Department of Medical and Surgical Sciences, Centro di Studio e Ricerca Sulle Neoplasie (CSR) Ginecologiche, Alma Mater Studiorum-University of Bologna, 40138 Bologna, Italy

**Keywords:** Imaging and sensing, Phase-contrast microscopy

## Abstract

Image-based identification of circulating tumor cells in microfluidic cytometry condition is one of the most challenging perspectives in the Liquid Biopsy scenario. Here we show a machine learning-powered tomographic phase imaging flow cytometry system capable to provide high-throughput 3D phase-contrast tomograms of each single cell. In fact, we show that discrimination of tumor cells against white blood cells is potentially achievable with the aid of artificial intelligence in a label-free flow-cyto-tomography method. We propose a hierarchical machine learning decision-maker, working on a set of features calculated from the 3D tomograms of the cells’ refractive index. We prove that 3D morphological features are adequately distinctive to identify tumor cells versus the white blood cell background in the first stage and, moreover, in recognizing the tumor type at the second decision step. Proof-of-concept experiments are shown, in which two different tumor cell lines, namely neuroblastoma cancer cells and ovarian cancer cells, are used against monocytes. The reported results allow claiming the identification of tumor cells with a success rate higher than 97% and with an accuracy over 97% in discriminating between the two cancer cell types, thus opening in a near future the route to a new Liquid Biopsy tool for detecting and classifying circulating tumor cells in blood by stain-free method.

## Introduction

The early diagnosis of a tumor condition can be considered nowadays the holy grail in cancer research, as it is crucial for improving the efficacy of therapeutic treatments. To date, the golden standard method for cancer diagnosis is the tissue biopsy, that is an invasive test involving the extraction of the cancerous tissue to be further analyzed^1^. However, this procedure only reflects the situation in a single site of the tumor at a single time instant, thus limiting the understanding of the complex characterization of a patient’s tumor. Indeed, it has been demonstrated that various areas within the tumor can harbor different genomic profiles^2^. Moreover, it takes long and cumbersome protocols. In principle, since tumors shed parts of themselves into the circulatory system, it is possible to detect them by analyzing body fluids, such as blood and urine. This approach, known as Liquid Biopsy (LB)^3–7^, provides a relatively less invasive methodology for detecting disease-derived biomarkers from body fluid samples, which make it an ideal method for routine evaluation. LB technologies are based on strategies for identifying and/or isolating biomarkers, such as circulating tumor DNA (ctDNA), tumor-derived exosomes (TDEs) and circulating tumor cells (CTCs)^7,8^. In particular, ctDNA can be extracted from the plasma by using columns, magnetic beads, polymer, phenol-chloroform-based and filtration-based methods^9^ and analyzed to search for cancer genomic alterations by droplet digital polymerase chain reaction (ddPCR), targeted deep sequencing (TD-Seq), whole exome sequencing (WES), whole genome sequencing (WGS) and whole genome bisulfite sequencing (WGBS-Seq), see citations^6,7,10^ and references therein for more details. TDEs isolation can be performed by centrifugation, size-based, capture-based, polymer-based, and microfluidics-based techniques^11^ and several technologies have been developed to analyze exosomes and exosomal cargoes^6^, including enzyme-linked immunosorbent assay (ELISA), flow cytometry and nanoparticle tracking analysis (NTA).

**Figure 1:**
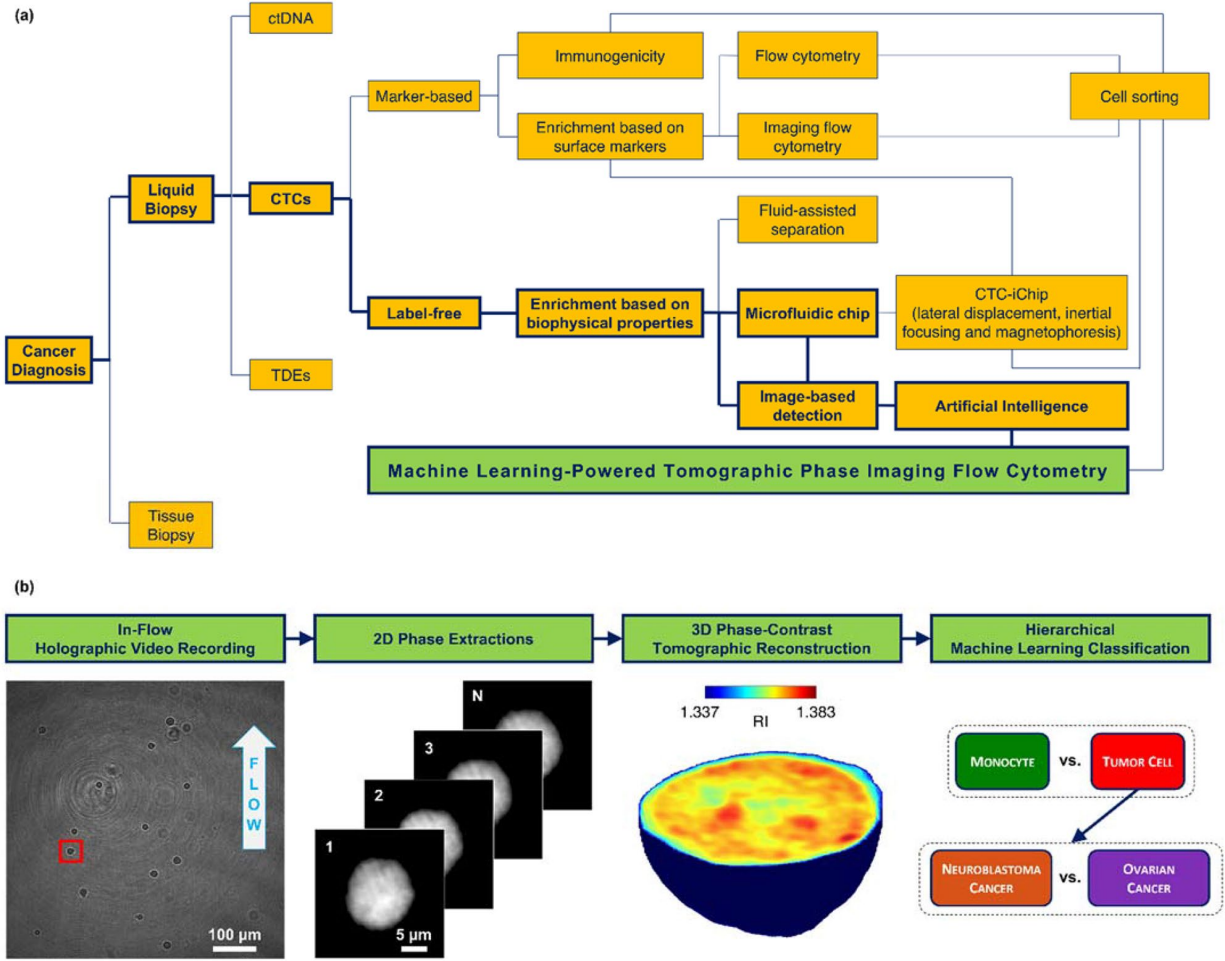
Strategies for implementing liquid biopsy (LB). (**a**) Scheme of the main methods developed for the diagnosis of cancer through LB. The marked pathway leads to the technology presented here for LB applications (green box). ctDNA, circulating tumor DNA; TDEs, tumor-derived exosomes; CTCs, circulating tumor cells. (**b**) Steps of the machine learning-powered tomographic phase imaging flow cytometry. (i) A holographic video sequence containing cells while flowing and rotating along a microfluidic channel is recorded; (ii) for each cell, hundreds of QPMs are numerically computed from the recorded digital holograms; (iii) for each cell, the several QPMs are used to reconstruct its 3D RI tomogram; (iv) the 3D RI tomogram is given in input to a hierarchical ML classifier to detect whether it is a monocyte or a tumor cell and, in the second case, whether it is a neuroblastoma cancer cell or an ovarian cancer cell.

However, in the last decade, an increasing attention has been focused on techniques for the detection and isolation of the CTCs^12–14^, including label-free approaches and microfluidic inspection systems^15–17^. Recently, new perspectives have been foreseen in finding new strategies based on intelligent lab-on-a-chip for detecting tumor cells^18^. In particular, it is possible to identify and then isolate these cells from the blood with high-throughput and afterwards conduct assays at the single-cell level^19^. Moreover, CTCs can provide crucial information for setting a precise, dynamic, and treatment-related method to tackle cancer over the course of the disease or the treatment itself ^19^. The current well-established technologies to isolate CTCs are based on immunogenicity, positive enrichment, negative enrichment, enrichment based on biophysical properties (i.e., size, deformability, density)^20^. In particular, the latter approach enables label-free methodologies since the isolation is basically dependent on the morphometric differences between CTCs and blood cells. Currently, using whole blood samples presents some challenges for size-based microfluidic methods, mainly due to membrane clogging related to the very high concentration of blood cells^20^. To overcome this problem, several devices have been recently developed based on various microfluidic chip designs^20,21^ or exploiting fluid-assisted separation technologies^22^. One of the most accurate systems of CTCs isolation is the CTC-iChip^23^ that combines three technologies to separate CTCs from whole blood exploiting different biophysical parameters. The CTC-iChip uses deterministic lateral displacement, inertial focusing and magnetophoresis to sort up to 10^7^ cells/s, achieving a 97% yield of rare cells with a sample processing rate of 8 ml of whole blood per hour^23^. However, in all these strategies/techniques, including the CTC-iChip, some residual white blood cells (WBCs) with biophysical features similar to the CTCs, are collected. Therefore, a subsequent analysis is necessary not only to identify the origin tumor tissue of the cancer cells but also for their effective count. To further push forward the performance of CTCs detection and isolation technologies, a way may be represented by the integration of artificial intelligence (AI), which has been recognized as the missing milestone to achieve very accurate single-cell analysis^24,25^. In the context of CTCs identification, AI-powered image-based systems have shown to be highly effective in classifying CTCs with respect to a background of WBCs^26,27^. Figure 1a contains a conceptual scheme of the various possibilities offered for the cancer diagnosis at the actual state of the art.

Since these approaches are based on the classification of single cells images, their performances are strictly related to the ability of the chosen imaging modality to encode the distinctive biophysical information allowing for the accurate identification of the CTCs with respect to other blood cells. Among the possible microscopy solutions, the label-free and quantitative phase imaging (QPI) by digital holography (DH) is one of the most informative^28^. QPI allows accessing the whole morphological information of cells, in analogy to the brightfield imaging or light scattering, and the intracellular organization of organelles thanks to the endogenous marker represented by the refractive index (RI). This enables to bypass the need of chemical stains typical of fluorescence microscopy. Furthermore, a label-free approach allows overcoming the main limitations of the marker-based strategies, i.e. the a priori knowledge of the exact protein composition on the CTCs surfaces and the lack of universal markers able to identify all heterogeneous CTCs in the bloodstream^15^. Tomographic phase imaging (TPI) is the latest cutting-edge QPI-based microscopy system, in which the volumetric RI distribution is achieved by probing the imaged cells from different directions^29–31^, thus allowing a 3D, quantitative and label-free characterization of the cell’s biophysical properties^32^. AI-based approaches have been recently fed by 3D RI tomograms recorded through a static TPI system for classifying different WBC types^33^, for identifying various lymphocyte species^34^, and for the screening of hematologic disorders by the analysis of red blood cells^35^. The recent demonstration of TPI in flow cytometry (TPI-FC) condition^36–38^ promises to bridge gaps with respect to the conventional imaging flow cytometry systems, i.e. allowing comparable throughput with potentially video-rate data processing^39–42^ and guaranteeing the intracellular specificity as in the fluorescence microscopy^43–46^. Moreover, TPI-FC has been already demonstrated to be very powerful in characterizing tumor cells^42,43,45–47^. Very recently, a first proof of the powerful applications derived by the combination between AI and TPI-FC has been given by recognizing the chemotherapy resistance in endometrial cancer cells^48^.

Here we present an AI-powered TPI-FC as a new methodology for LB, as sketched in the marked pathway in Figure 1a. Such method has potential high-throughput capability in retrieving phase-contrast tomograms of single cells in continuous flow. Essentially, as sketched in Figure 1b, here we demonstrate that, by recording the digital holograms of single cells while flowing and rotating along a microfluidic channel and by retrieving their 2D phase-contrast maps, it is possible to collect a dataset of 3D RI tomograms to be analyzed by a hierarchical machine learning (ML) decision-maker. The aim is to discriminate between WBCs and tumor cells at the first stage and then recognize the type of tumor at the second decision level. As a proof of concept, we show an image-based detection technique for identification of two cell lines, namely neuroblastoma (NB) cancer cells and ovarian cancer (OC) cells, against monocytes (MCs). In fact, in terms of size, MCs are recognized to be the closest cells in human blood to tumor cells. It is important to highlight that our system is thought out to be integrated into existing technologies, in which, as first step, the whole blood needs to be filtered to remove platelets and red blood cells (e.g. by using the deterministic lateral displacement as in the CTC-iChip). As second step, in our approach, the intelligent tomograms-based decision is taken and a sorting module is activated by the above decision, with the aim to collect only the identified CTCs. The idea follows a conceptual workflow similar to the so-called Intelligent Image-Activated Cell Sorter (iIACS)^49^. The results attained here show a tumor cells identification success > 97 % along with an accuracy > 97 % in discriminating between the two cancer cell types. Such challenging capability confirms, in principle, the feasibility of the proposed method in realistic scenario of future LB by stain-free mode.

## Results

### Tomographic dataset and 3D data augmentation

**Figure 2:**
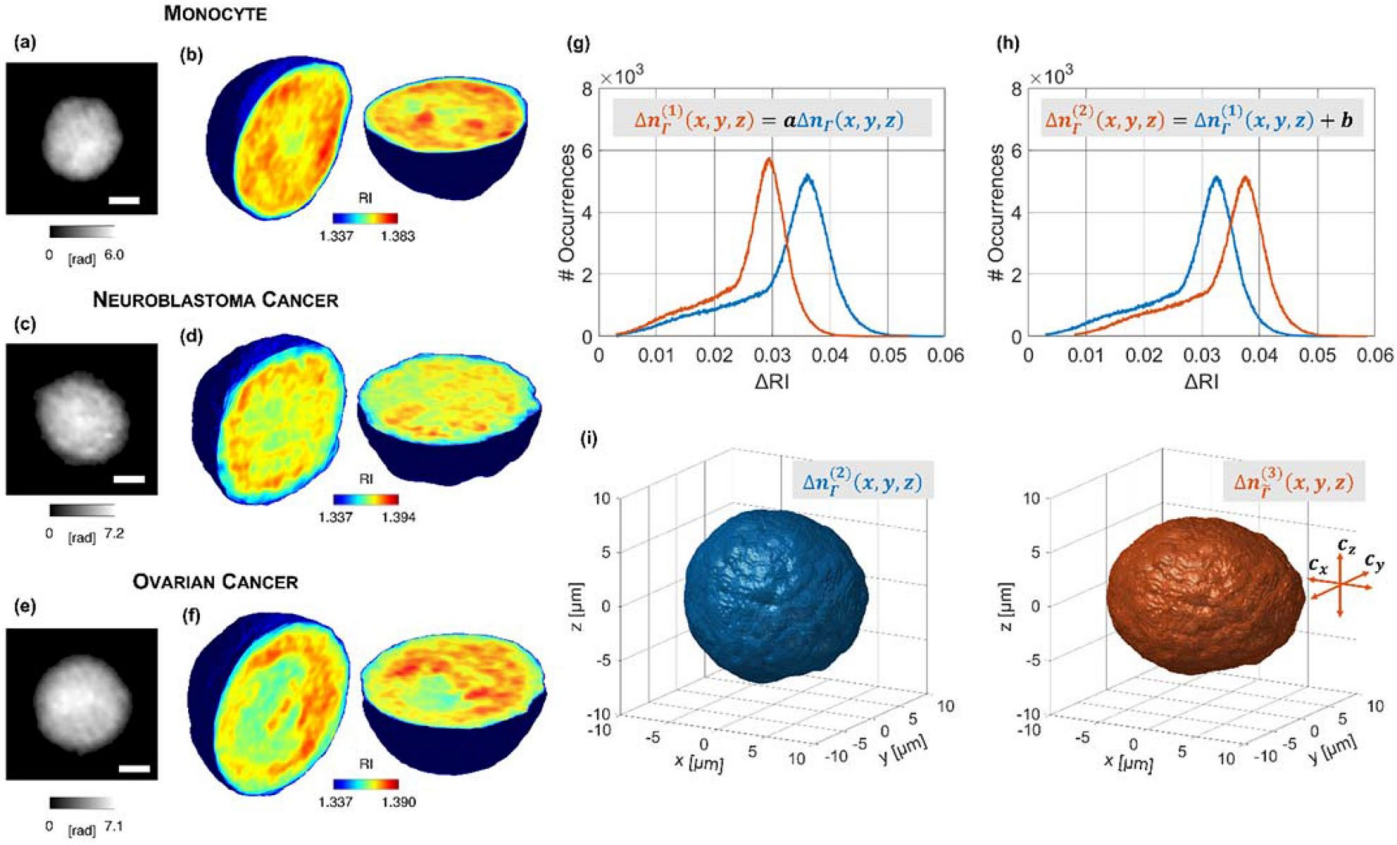
Tomographic dataset collected through the TPI-FC system and augmented for ML training, containing (**a**,**b**) MC cells (THP1 cell in this example), (**c**,**d**) NB cells (SKNSH cell in this example), and (**e**,**f**) OC cells (CAOV3 in this example). (**a**,**c**,**e)** QPM at 0° of rotation. The scale bar is 5 µm. (**b**,**d**,**f**) Central slices of the 3D RI tomograms before the 3D data augmentation. (**g**) Intensity scaling of the RI values (after subtracting ${n}_{0}$) belonging to the SKNSH tomogram in (**b**). $a=0.9$ in this example. (**h**) Intensity shifting of the RI values (after subtracting ${n}_{0}$) belonging to the SKNSH tomogram in (**b**) after the intensity scaling in (**g**). $b=0.005$ in this example. (**i**) On the right, 3D shape of the augmented tomogram after the morphological alteration of the 3D shape on the left corresponding to the SKNSH tomogram in (**b**). ${c}_{x}=20$, ${c}_{y}=0$, ${c}_{z}=-20$ in this example, hence the tomogram is stretched along the $x$-axis and compressed along the $z$-axis.

The TPI-FC experimental setup and the corresponding numerical processing described in the “Methods” section have been used for the experiments and for retrieving the reported tomographic dataset. The dataset has been used for training a hierarchical ML model at the aim of identifying first tumor cells from MCs and then recognizing the type of tumor. In particular, hundreds of digital holograms of each single cell were recorded while the cells were flowing and rotating along a microfluidic channel. From each digital hologram, the corresponding quantitative phase map (QPM) is numerically extracted. In a QPM, the information about the 3D spatial distribution of the cell’s RIs is coupled to the information about the 3D morphology of the cell in the form of a 2D image. Therefore, to decouple them, after retrieving the corresponding unknown rolling angles, the QPMs are combined to reconstruct the 3D RI tomogram at the single-cell level. Following this pipeline, the 3D RI tomograms of several cell lines have been reconstructed, as summarized in Table S1. In particular, (1) as regards the MC class, 247 THP1 cells have been recorded, (2) as regards the NB class, 372 cells have been recorded, i.e. 115 CHP212 cells, 106 SKNBE2 cells, and 151 SKNSH cells, (3) as regards the OC class, 310 cells have been recorded, i.e. 95 A2780 cells and 215 CAOV3 cells. In Fig. 2a,c,e, an example of QPM is reported for a MC cell (THP1), a NB cell (SKNSH), and an OC cell (CAOV3), respectively, while the central slices of the corresponding 3D RI tomograms are shown in Fig. 2b,d,f, respectively. For these three cells, the overall sequence of QPMs and their 3D RI reconstructions (both a slice-by-slice visualization and an isolevels representation) can be seen in the Supplementary Movie S1. To train and test a ML model, the tomographic dataset has been divided into a training set containing 700 cells (200 MC, 250 NB, and 250 OC cells) and a test set containing 229 cells (47 MC, 122 NB, and 60 OC cells), as reported in Table S1. To counteract the limited sizes of our datasets, the training set has undergone a 3D data augmentation process. In particular, 9×, 3× and 3× augmented tomograms have been computed from each MC, NB, and OC cells respectively. Therefore, as shown in Table S1, the augmented tomographic dataset is made of 2000 MC, 1000 NB, and 1000 OC cells. To perform the 3D data augmentation, three successive operations have been applied to each 3D RI tomogram, i.e. (1) intensity scaling, (2) intensity shifting, (3) morphological alteration. The 3D data augmentation, as well as the holographic processing, the tomographic reconstructions, the feature extraction, and the ML training have been carried out by means of MATLAB® R2022b. Let $n\left(x,y,z\right)$ be the RI volumetric distribution inside the ${L}_{x}\times {L}_{y}\times {L}_{z}$ array containing the cell (in our TPI-FC system, ${L}_{x}={L}_{y}={L}_{z}=201$ pixels), and let ${n}_{0}$ be the RI of the surrounding medium, supposed homogeneous. Let $\Gamma$ be the volumetric support of the cell, i.e. the set of voxels $\left(x,y,z\right)$ occupied by the cell, and let ${n}_{\Gamma }\left(x,y,z\right)$ be the set of cell’s RI values. In our experiments, ${n}_{0}=1.334$ and ${\Delta n}_{\Gamma }\left(x,y,z\right)>0$, where ${\Delta n}_{\Gamma }\left(x,y,z\right)={n}_{\Gamma }\left(x,y,z\right)-{n}_{0}$.

1. As regards the intensity scaling, the ${\Delta n}_{\Gamma }\left(x,y,z\right)$ values are rescaled by a factor $a$ randomly drawn from the uniform distribution $U\left(\mathrm{0.9,1.1}\right)$, thus obtaining the $\Delta {n}_{\Gamma }^{(1)}\left(x,y,z\right)$ values.
2. As regards the intensity shifting, the $\Delta {n}_{\Gamma }^{(1)}\left(x,y,z\right)$ values are shifted by a factor $b$ randomly drawn from the uniform distribution $U\left(-min\left(\Delta {n}_{\Gamma }^{(1)}\left(x,y,z\right)\right)/2,min\left(\Delta {n}_{\Gamma }^{(1)}\left(x,y,z\right)\right)/2\right)$, thus obtaining the $\Delta {n}_{\Gamma }^{(2)}\left(x,y,z\right)$ values ($min$ is the operator that calculates the minimum value of a function).
3. As regards the morphological alteration, the ${L}_{x}\times {L}_{y}\times {L}_{z}$ volume is rescaled into the $\left({L}_{x}+{c}_{x}\right)\times \left({L}_{y}+{c}_{y}\right)\times \left({L}_{z}+{c}_{z}\right)$ volume through the *imresize* MATLAB function, where ${c}_{x}$, ${c}_{y}$ and ${c}_{z}$ are separately randomly drawn from the uniform distribution $U\left(-\mathrm{20,20}\right)$, thus obtaining the $\Delta {n}_{\widetilde{\Gamma }}^{(3)}\left(x,y,z\right)$ values ($\widetilde{\Gamma }$ is the new volumetric support of the cell after the morphological alteration). Hence, for example, if ${c}_{x}>0$, the tomogram is stretched along the $x$-axis, otherwise, if ${c}_{x}<0$, the tomogram is compressed along the $x$-axis.

After these three operations, the augmented 3D RI tomogram is obtained as $\widetilde{n}\left(x,y,z\right)=\Delta {n}^{(3)}\left(x,y,z\right)+{n}_{0}$. An example of intensity scaling, intensity shifting, and morphological alteration is reported in Fig. 2g–i, respectively, based on the 3D RI tomogram of the SKNSH cell in Fig. 2d.

### Features extraction

**Figure 3:**
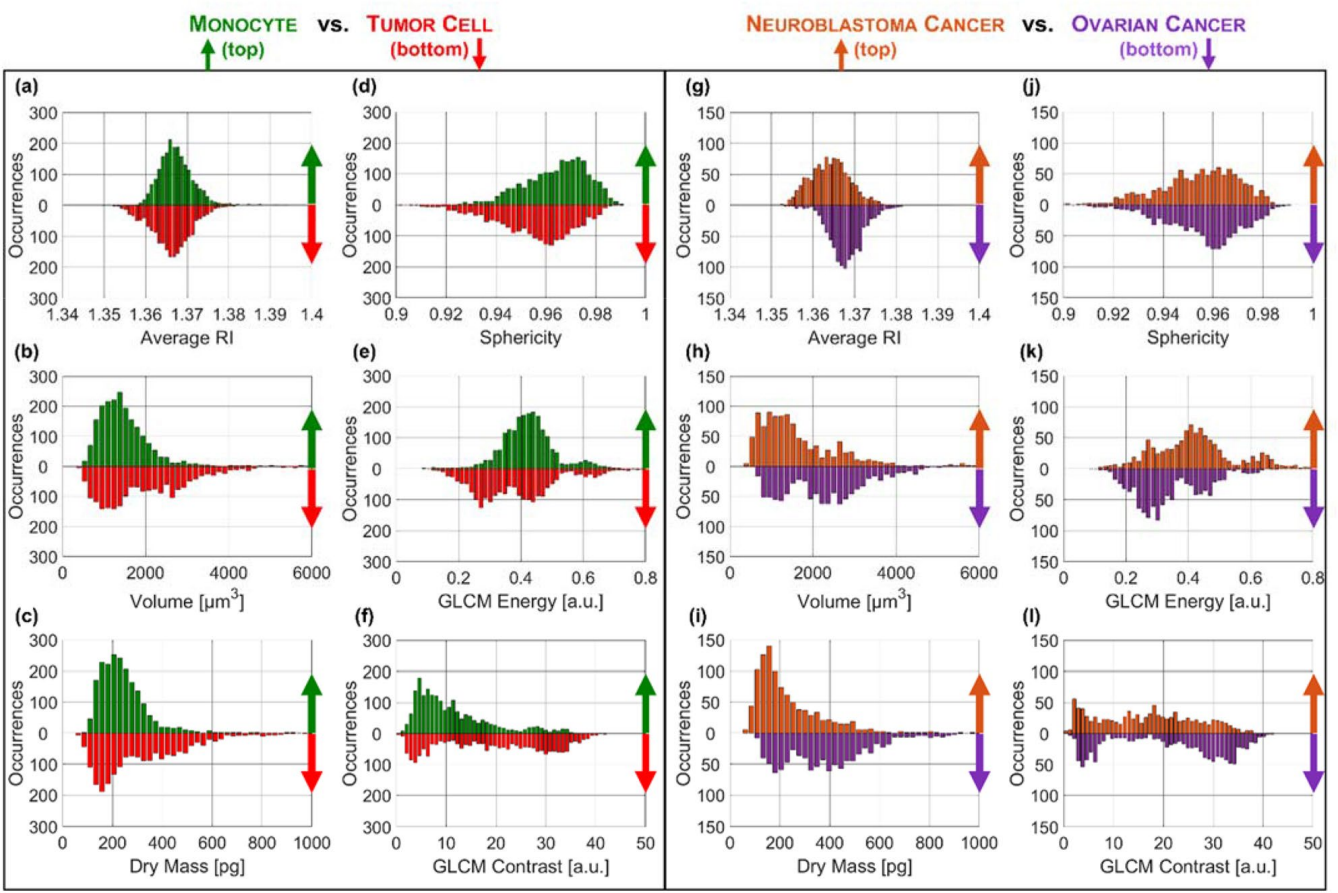
Feature set measured from the augmented training set made of 3D RI tomograms. (**a**–**f**) Histograms of the average RI, volume, dry mass, sphericity, GLCM energy, and GLCM contrast, respectively, related to the MCs (top) vs. tumor cells (bottom) problem. (**g**–**l**) Histograms of the average RI, volume, dry mass, sphericity, GLCM energy, and GLCM contrast, respectively, related to the NB (top) versus OC (bottom) problem.

In order to train a ML model, 44 features have been calculated from each 3D RI tomogram. In particular, 11 features are related to the RI statistical distribution: the average, the median, the mode, the maximum, the standard deviation, the skewness, the entropy, the kurtosis, the 0.25-quantile, the 0.75-quantile, and the dry mass (defined as the mass of the cell without considering its aqueous content^50^). Other 9 features are instead related to the 3D morphology of the cell, such as the volume, the convex volume, the sphericity, the extent, the solidity, the lengths of the three principal axes, and the normalized centroids’ distance. The convex volume is the volume of the smallest convex polygon containing the cell. The sphericity is the ratio between the surface area of a sphere with the same volume of the cell and the surface area of the cell (it is 1 for spherical objects, otherwise it is less than 1). The extent is the ratio between the cells volume and the volume of the cells bounding box, i.e. the smallest cuboid containing the cell. The solidity is the ratio between the volume and the convex volume. The principal axes are the major axes of the ellipsoid having the same normalized second central moments as the cell. The normalized centroids’ distance is the distance between the centroid and the weighted centroid (with respect to the RI volumetric distribution) of the cell, normalized to the cells equivalent radius, defined as the radius of a sphere having the same volume as the cell. The morphological features have been computed by means of the *regionprops3* MATLAB function. Finally, 24 features have been computed as the Haralick features of the 3D Gray-Level Co-Occurrence Matrix (GLCM)^51^. The GLCM describes the different combinations of the grey levels within an image. Indeed, the GLCM $G(h,k,\left[{\widehat{i}}_{x},{\widehat{i}}_{y},{\widehat{i}}_{z}\right],d)$ measures how many times a pixel with value $h$ occurs along the direction $\left[{\widehat{i}}_{x},{\widehat{i}}_{y},{\widehat{i}}_{z}\right]$ at distance $d$ with respect to a pixel with value $k$. Therefore, after fixing $d=0.5$ μm, for each of 13 3D directions ($\left[\mathrm{0,1},0\right]$, $\left[-\mathrm{1,1},0\right]$, $\left[-\mathrm{1,0},0\right]$, $\left[-1,-\mathrm{1,0}\right]$, $\left[\mathrm{0,1},-1\right]$, $\left[\mathrm{0,0},-1\right]$, $\left[0,-1,-1\right]$, $\left[-\mathrm{1,0},-1\right]$, $\left[\mathrm{1,0},-1\right]$, $\left[-\mathrm{1,1},-1\right]$, $\left[1,-1,-1\right]$, $\left[-1,-1,-1\right]$, and $\left[\mathrm{1,1},-1\right]$) the 12 Haralick features have been computed^52^, i.e. energy, entropy, correlation, contrast, variance, sum average, inertia, cluster shade, cluster tendency, homogeneity, maximum probability, and inverse variance. The 12 Haralick features at $d=0.5$ μm have been finally averaged among the 13 3D directions, thus obtaining 12 of the 24 GLCM features. Instead, the other 12 GLCM features have been computed in the same way by fixing $d=1$ μm. The histograms of some features computed from the 3D RI tomograms of the augmented training set are displayed in Fig. 3a–l. In particular, in Fig. 3a–f, the average RI, the volume, the dry mass, the sphericity, the GLCM energy, and the GLCM contrast are shown by separating the MCs from the cancer cells (NB and OC cells). Instead, the same features are reported in Fig. 3g–l, respectively, by separating the NB cells from the OC cells. For example, it can be inferred that the sphericity histogram of the tumor cells is left-shifted with respect to that of the MCs (see Fig. 3d), while the average RI histogram of the OC cells is right-shifted with respect to that of the NB cells (see Fig. 3g). Furthermore, in the following Section, results of the ML classification based on the 3D RI tomograms will be compared with the ML classification based on the 2D QPMs of the same cells. Therefore, 44 features like those described above have been measured from each QPM. The 11 features related to the phase statistical distribution are again the average, the median, the mode, the maximum, the standard deviation, the skewness, the entropy, the kurtosis, the 0.25-quantile, the 0.75-quantile, and the dry mass^28^. The 9 2D morphological features are the area, the extent, the solidity, the circularity, the eccentricity, the maximum and minimum Feret diameters, the major axis length, and the normalized centroids’ distance, computed by means of the *regionprops* MATLAB function. The circularity is computed as $4A\pi /{P}^{2}$, where $A$ and $P$ are the area and the perimeter of the cell, respectively, and it is 1 for a perfect circle. The major axis length is the length of the major axis of the ellipse having the same normalized second central moments as the cell, while the eccentricity is the ratio of the distance between the foci of the same ellipse and the major axis length. The maximum and minimum Feret diameters are respectively the maximum and minimum distances between any two boundary points on the antipodal vertices of the convex hull enclosing the cell. The normalized centroids’ distance is the distance between the centroid and the weighted centroid (with respect to the phase spatial distribution) of the cell, normalized to the cell’s equivalent radius, defined as the radius of a circle having the same area as the cell. Finally, 12 2D GLCM features have been computed by averaging the 12 Haralick features along the $\left[\mathrm{0,1},0\right]$, $\left[-\mathrm{1,1},0\right]$, $\left[-\mathrm{1,0},0\right]$, and $\left[-1,-\mathrm{1,0}\right]$ directions by fixing $d=0.5$ μm, while the other 12 2D GLCM features have been computed by considering $d=1$ μm.

### Hierarchical ML classification

**Figure 4:**
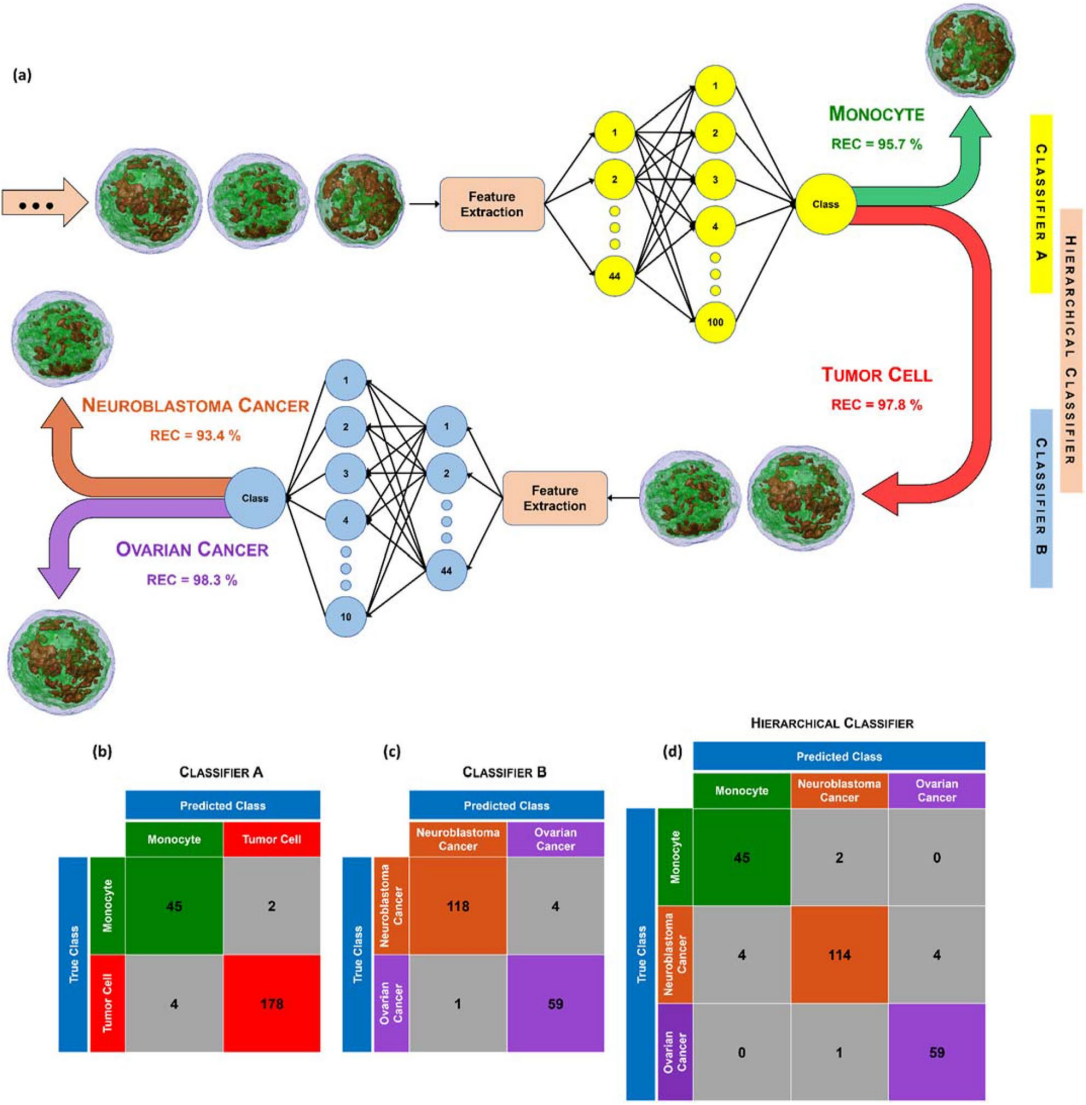
ML classification of 3D RI tomograms. (**a**) Conceptual scheme of the hierarchical model made of two successive shallow neural networks (classifier **A** and classifier **B**), with highlighted its recall values. The classifier **A** detects MCs vs. tumor cells. The classifier **B** recognizes NB cells vs. OC cells. The hierarchical classifier identifies MC cells versus NB cells versus OC cells. Cells are visualized through a 3-isolevels representation (the RI thresholds are set as the maximum RI value multiplied by 0.6 and 0.75). (**b**–**d**) Confusion matrices of the classifier **A**, classifier **B**, and hierarchical classifier, respectively.

Usually, in LB, CTCs are firstly detected inside a background of WBCs, and then the particular type of cancer is recognized. Therefore, following this scheme, we have built a hierarchical classifier made of two successive binary classifiers (namely, **A** and **B**). A shallow neural network with three layers has been chosen for both classifiers **A** and **B**, as sketched in Fig. 4a. The input layer is made of 44 nodes, corresponding to the 44 features described in the previous Section. The hidden layer is a fully convolutional layer made of 100 nodes for the classifier **A** and 10 nodes for the classifier **B**. The output layer is made of 1 node which provides the predicted class. The ReLU is selected as activation function. By considering the augmented dataset of 3D RI tomograms summarized in Table S1, the classifier **A** has been trained through 2000 MCs and 2000 cancer cells (1000 NB cells and 1000 OC cells), while the classifier **B** has been trained through 1000 NB and 1000 OC cells. To test the hierarchical classifier, 229 cells (47 MC, 122 NB, and 60 OC cells) have been employed (see Table S1). The confusion matrices related to the classifier **A** (MCs vs. tumor cells), the classifier **B** (NB cells vs. OC cells), and the overall hierarchical classifier (MCs vs. NB cells vs. OC cells) are reported in Fig. 4b–d, respectively, while the corresponding classification metrics are summarized in Tables 1 and 2 (the mathematical definitions can be found in the Supplementary Information). Given a certain class, let the recall (REC) be the percentage of elements belonging to that class which are correctly classified. If the classifiers **A** and **B** are considered separately, the classifier **A** detects the MC class with $REC=95.7\%$ and the tumor cell class (NB cells or OC cells) with $REC=97.8\%$ (Fig. 4b and Table 1), while the classifier **B** recognizes the NB class with $REC=96.7\%$ and the OC class with $REC=98.3\%$ (Fig. 4c and Table 1). However, within the hierarchical classifier, the misclassification error propagates from the input of the classifier **A** to the output of the classifier **B**. Hence, after extracting the 44 features from the 3D RI tomograms of the single-cells recorded through the TPI-FC system, they are given as input of the classifier **A**, which detects a MC or a tumor cell with a certain error. The 44 features of the sole cells classified as tumor cells at the output of the classifier **A** are given as input of the classifier **B**, which at the end recognizes the type of cancer (i.e. NB or OC) with its additional error. Therefore, the hierarchical classifier detects the MC cells with $REC=95.7\%$, the NB cells with $REC=93.4\%$, and the OC cells with $REC=98.3\%$ (Fig. 4d and Table 2). To measure the overall performance of a classifier, we considered the accuracy (ACC), that is the probability of correctly classifying an element given as input of the model. The classifier **A** has $ACC=97.4\%$ (Fig. 4b and Table 1), the classifier **B** has $ACC=97.3\%$ (Fig. 4c and Table 1), and the overall hierarchical classifier has $ACC=95.2\%$ (Fig. 4d and Table 2). Hence, the combination proposed here between the TPI-FC data and a ML hierarchical classifier offers the possibility for recognizing and then phenotyping cancer cells with very high accuracy. This is mainly due to the ability of TPI-FC of providing a statistical measurement of the distinctive fingerprint of the single cells belonging to a certain population (thanks to the FC module) because their biophysical content can be accessed in 3D (thanks to the TPI module). However, the tomographic reconstruction requires larger hardware resources and longer computational times if compared to a more conventional QPI in flow cytometry (QPI-FC) system, because hundreds of digital holograms per cell must be processed instead of just one digital hologram per cell. For this reason, a comparison of the classification performance achievable by a QPI-FC system is needed to justify the greater burden of a TPI-FC system. To this aim, to have a fair comparison between these two strategies, by using the straight-ray model of the light propagation (i.e., the same model used by the employed tomographic reconstruction code), one QPM per cell has been computed by integrating at $0^\circ$ each 3D RI tomogram belonging to the augmented training set and to the test set described in Table S1. Then, the hierarchical classifier has been trained by means of the 44 features measured from the 2D QPMs. In this case, the logistic regression has been chosen as classifier **A** and the linear discriminant has been chosen as classifier **B**, since they provided the best test performance (see Tables 1 and 2). In particular, the classifier **A** has $ACC=87.3\%$ (instead of the 3D $ACC=97.4\%$, see Table 1), the classifier **B** has $ACC=85.2\%$ (instead of the 3D $ACC=97.3\%$, see Table 1), and the overall hierarchical classifier has $ACC=76.0\%$ (instead of the 3D $ACC=95.2\%$, see Table 2). Moreover, as reported in Table 2, the hierarchical classifier detects the MC cells with $REC=63.8\%$ (instead of the 3D $REC=95.7\%$), the NB cells with $REC=77.9\%$ (instead of the 3D $REC=93.4\%$), and the OC cells with $REC=81.7\%$ (instead of the 3D $REC=98.3\%$). In summary, all the performance given by a QPI-FC system are drastically lower than the TPI-FC method, as summarized by the accuracy of the hierarchical classifier, which decreases by $19.2\%$.

**Table 1:** Performance of the classifier A (shallow neural network in the case of 3D RI tomograms and logistic regression in the case of 2D QPMs) and the classifier B (shallow neural network in the case of 3D RI tomograms and linear discriminant in the case of 2D QPMs) over the test set.

Metric	Classifier A				Classifier B			
	3D RI tomogram		2D QPM		3D RI tomogram		2D QPM	
	MC	Tumor cell	NB	OC	NB	OC	MC	Tumor cell
Accuracy	97.4		87.3		97.3		85.2	
True positive rate (sensitivity or recall)	95.7	97.8	96.7	98.3	82.8	90.0	63.8	93.4
True negative rate (specificity)	97.8	95.7	98.3	96.7	90.0	82.8	93.4	63.8
Positive predictive value (precision)	91.8	98.9	99.2	93.7	94.4	72.0	71.4	90.9
Negative predictive value	98.9	91.8	93.7	99.2	72.0	94.4	90.9	71.4
Balanced accuracy	96.8		78.6		97.5		86.4	
F1 score	93.8	98.3	97.9	95.9	88.2	86.4	67.4	92.1
Matthews correlation coefficient	92.1		59.7		93.9		69.5	
Fowlkes–Mallows index	93.8	98.3	97.9	96.0	88.4	80.5	67.5	92.1

**Table 2:** Performance of the hierarchical classifier over the test set (shallow neural network + shallow neural network in the case of 3D RI tomograms and logistic regression + linear discriminant in the case of 2D QPMs).

Metric	3D RI Tomogram			2D QPM		
	MC	NB	OC	MC	NB	OC
Accuracy	95.2			76.0		
True positive rate (sensitivity or recall)	95.7	93.4	98.3	63.8	77.9	81.7
True negative rate (specificity)	97.8	97.2	97.6	93.4	86.9	82.8
Positive predictive value (precision)	91.8	97.4	93.7	71.4	89.2	62.8
Negative predictive value	98.9	92.9	99.4	90.9	77.5	92.7
Balanced accuracy	96.8	95.3	98.0	78.6	82.4	82.2
F1 score	93.8	95.4	95.9	67.4	82.3	71.0
Matthews correlation Coefficient	92.1	90.5	94.5	59.7	64.7	59.9
Fowlkes–Mallows index	93.8	95.4	96.0	67.5	82.4	71.6

## Discussion

The proposed methodology based on the ML-powered TPI-FC has been demonstrated to be very efficient in identifying tumor cells with respect to the background of MCs, also providing the possibility to discriminate between two cancer cell lines, i.e. NB and OC. While only a proof-of-concept study has been addressed in this paper, it is reasonable to foresee that the extension of the approach to the identification of other cancer cell lines, as well as other types of WBCs, is feasible. In this case, it is needed to consider a more heterogeneous population of WBCs and CTCs. Moreover, for the cancer cells, further levels of classification could be included for the phenotyping of CTCs, thus discriminating among sub-types. On the other hand, the choice of using only MCs as the WBC population has two main motivations. First, it has been demonstrated that MCs may play an important role in metastasis, and they can extravasate and differentiate into macrophages, promoting tumor cell extravasation, survival, and subsequent growth^53^. Therefore, the possibility to analyze MCs in combination with cancer cells can provide further information about tumor treatments. Second, MCs are often comparable in size with respect to CTCs, thus making a challenge their preliminary separation from the whole blood sample. In fact, since the intent of the proposed approach would be the integration in existing technologies that provide the removal of platelets, red blood cells and smaller WBCs, there is high probability to collect MCs and CTCs together. Our results show that ML-powered TPI-FC can correctly identify tumor cells with respect to MCs with an accuracy > 97 %, thus extremely limiting this scenario. In addition, the possibility to use the classification output as the actuator signal in an intelligent image-activate sorter further confirms the ability to increase the CTCs enrichment effectiveness. However, to clearly understand the potentiality of the proposed method in a real LB application, it would be valuable to quantify the device performance using well-recognized parameters^54^ such as capture efficiency, enrichment, purity, throughput, cell viability, and release efficiency. By considering the experiments carried out in this paper, we can define the purity as the probability of correctly recognizing a tumor cell with respect to a background of interfering cells, i.e. the MCs, that is 97.8 % (i.e., the recall parameter). The cell viability is expected to be very high in our label-free system, and cells can be used again for further downstream analyses, mainly represented by high-throughput sequencing approaches, that allow us searching in unbiased and large-scale manner for different cancer genomic alterations, or approaches for mutation specific detection. As regards the throughput, we have already demonstrated that, for a Field-of-View (FoV) = 160 µm × 215 µm, the holographic data for reconstructing an upper bound of about 50/60 tomograms/s can be recorded^41^. Hence, in the current FoV = 640 µm × 640 µm, this value can be increased of about 10 times. Moreover, the release efficiency, which describes the number of cells that are recovered by the system, should be related to the ability of the system in providing a video-rate processing and classification of the tomograms. Finally, the capture efficiency and enrichment, which are related to the ability of the system in capturing CTCs from a sample, cannot be defined at this stage since, as stated above, we have considered already done the pre-filtration of the blood sample. In summary, identification of CTCs, reflecting intra-tumoral heterogeneity, represent a promising pathway for cancer diagnosis and monitoring although the current unavailability of universal and specific cell-surface markers makes CTCs detection a challenging task. In this context, we have proposed a ML-powered TPI-FC system aimed to discriminate tumour cells within a blood cells background and to catch their phenotypic heterogeneity. Our results suggest that this label-free approach for rapid and efficient phenotyping of cancer cells could represent a suitable tool for the identification of novel morphological biomarkers to discriminate CTCs and to distinguish clinically aggressive from more favourable CTCs.

## Methods

### Sample preparation

The human CHP212, SKNBE2, and SKNSH NB cell lines were obtained from the American Type Culture Collection (respectively ATCC #CRL-2273, # CRL-2271, and #HTB-11). CHP212 cells were grown in Minimal Essential Eagle Medium (MEM; Sigma)/Nutrient Mixture F-12 (F-12), SKNBE2 in Dulbecco's Modified Eagle Medium (DMEM; Sigma)/F-12, and SKNSH in MEM at 37 °C, 5% CO2 in a humidified atmosphere. The medium was supplemented with 10% heat-inactivated FBS (Sigma), 1 mmol/L L-glutamine, penicillin (100 U/mL), and streptomycin (100mg/mL; Invitrogen). The cell lines were authenticated and early-passage cells were used for all the experiments. The human OC A2780 and CAOV3 cell lines were purchased from Sigma Aldrich-MERCK (#93112519) and ATCC (#ATCC-HTB-75), respectively. Both OC cells were grown as a monolayer and cultured in RPMI 1640 Medium (Life technologies #31870-025) supplemented with 10% FBS (Life Technologies #10270), 1% Penicillin/Streptomycin (Life Technologies #15070-063) and 2mM L-Glutamine (Lonza BE #17-605E). They were cultivated at 37 °C in an incubator with 5% CO2. The human MC cell line THP1 was supplied by a third part and the cell origin was authenticated using AMPFlSTRIdentifiler kit (Applied Biosystems #4322288, data available on request). THP1 was cultured in suspension in 75 cm^2^ tissue-culture flasks (Corning, #353018), grown in RPMI 1640 Medium (Life Technologies, #31870-025), supplemented with 10% FBS (Life Technologies #10270), 2mM L-Glutamine (Lonza, Cat #BE17-605E) and 1% Penicillin/Streptomycin (Lonza, Cat #DE17-602E), and maintained at 37 °C in a humidified atmosphere with 5% CO2. To obtain cell suspensions for the TPI-FC experiments, THP-1 cells were harvested from the cell culture flask, centrifuged for 5 min at 1500 rpm and resuspended in PBS solution containing 10% FBS (10 mL). The NB and OC cells were detached following standard protocols. In particular, the cells were seeded in a T75 flask (Corning, #353018) to reach 70–90% confluency. On the day of the experiment. the medium was removed, the cells were washed twice with PBS (10 mL for each wash) and then incubated for 5 min at 37 °C with Trypsin-EDTA solution (2 mL, Sigma, #T4049). Subsequently, PBS containing 10% FBS (8 mL) was added to block trypsin activity. For all cell lines, prior to the experiment the viability was assessed using Trypan Blue dye (Sigma #T8154) following manufacturer’s instructions. In detail, the cell suspension (0.05 mL) was diluted 1:2 with Trypan Blue dye and 0.01 mL was loaded in the Burker counting chamber to count dead (blue) and live (transparent) cells. Cell populations with minimum 98% of live cells were used to prepare suspensions of 4 × 10^5^ cells/mL in PBS solution containing 10% FBS and 300 µL were injected into the microfluidic channel.

### TPI-FC system

**Figure 5:**
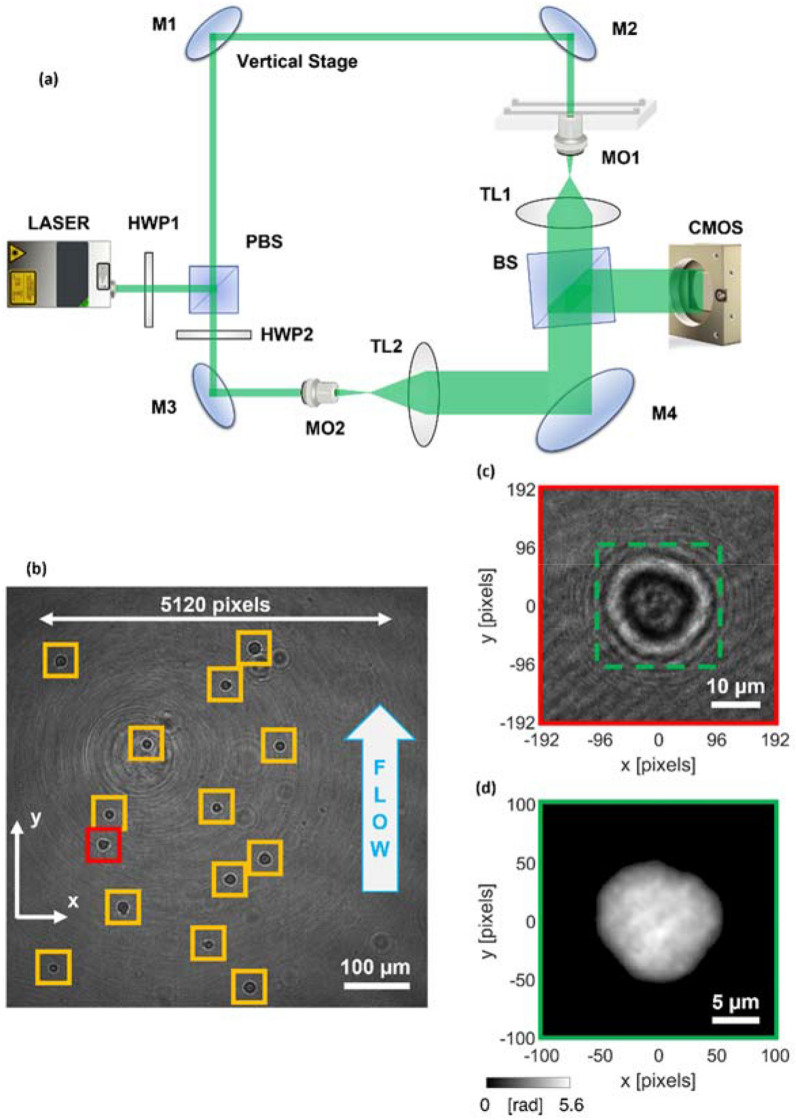
TPI-FC. (**a)** Opto-fluidic recording system, made of a DH microscope in off-axis configuration combined to a microfluidic module. HWP, half wave plate; MO, microscope objective; M, mirror; PBS, polarizing beam splitter; BS, beam splitter; TL, tube lens; CMOS, camera. (**b**) Digital hologram taken from the holographic video sequence recorded by the system in (**a**). The 384 × 384 holographic ROIs containing the cells are highlighted. (**c**) Zoom-in of the red holographic ROI in (**b**). (**d**) 201 × 201 QPM numerically extracted from the holographic ROI in (**c**).

The opto-fluidic recording system sketched in Fig. 5a has been employed for the TPI-FC experiments^40^. The optical module is an off-axis DH microscope based on a Mach–Zehnder interferometer. The beam generated by a coherent laser source (Laser Quantum Torus emitting at 532 nm) is divided into an object and a reference beam by a Polarizing Beam Splitter (PBS). Two Half-Wave Plates (HWPs) are used to balance the ratio between the two beams’ intensities. After illuminating the cells flowing inside the microfluidic channel, the object beam is collected by a high numerical aperture microscope objective (MO1, Zeiss Plan-ApoChromat 40×, NA = 1.3) and sent to a tube lens (TL1). Then, a Beam Splitter (BS) takes in input the object beam and the reference beam, which has passed in the meantime through a beam expander, a second microscope objective (MO2) and a second tube lens (TL2). The BS produces the interference between the two beams, properly collimated by the TLs, thus creating the digital hologram recorded at 30 fps by the CMOS camera (Teledyne Dalsa Genie Nano-CXP 5120 × 5120 pixels, 4.5 µm/pixel, 80 fps) and stored by digital recording unit (IOindustries DVR, Core 2, Video Storage Modules 4 × 3840 GB). The fluidic module is composed of an automatic low-pressure pump (CETONI base 120 Nemesys) generating a laminar flow at about 50 nl/s inside a rectangular-cross-section microfluidic channel (Microfluidic ChipShop 10,000,107 – 200 μm × 1000 μm × 58.5 mm). Therefore, cells flow along the microchannel and contemporarily rotate due to the torque generated by the velocity gradient in the channel cross-section, which, in turn, is due to the parabolic velocity profile characterizing the laminar flow. Let $z$ be the optical axis and $y$ be the flow direction. Cells are mainly recorded in the center with respect to the $x$-axis and in the bottom with respect to the $z$-axis^52^. As the FoV measures 640 µm × 640 µm (5120 × 5120 pixels), hundreds of digital holograms per cell are recorded at 30 fps along different viewing angles. An example of full-FoV digital hologram is displayed in Fig. 5b. Despite the excellent performance achieved here, the throughput of the device can be further increased by optimizing the design and the flow conditions of the microfluidic module, which is the object of ongoing work.

### Holo-tomographic numerical processing

Each 5120 × 5120 digital hologram of the video sequence recorded by the TPI-FC system is numerically processed to obtain the QPMs of flowing single cells^28^. As shown in Fig. 5b,c, from each holographic frame, 384 × 384 region of interests (ROIs) are cropped around the cells. As the DH microscope is in off-axis configuration, each holographic ROI is demodulated by selecting the real diffraction order in the Fourier spectrum through a band-pass filter^56^. In DH, the focal plane of each cell can be recovered numerically after the experiment. As displayed in Fig. 5c, the holographic ROI (i.e., the 384 × 384 red box) is larger than the size of the single cell (i.e., the 201 × 201 dashed green box) in order to include its entire diffraction pattern. It is precisely the diffraction pattern that allows implementing an automatic strategy for refocusing the cell (namely, autofocusing)^57^. In fact, the cell’s diffraction pattern changes when the demodulated holographic ROI is numerically propagated along the optical z-axis through the Angular Spectrum method^56^. For each z-distance, this variation can be quantified as the image contrast of the complex wavefront’s amplitude, measured through the Tamura Coefficient (TC). The focal distance of each cell is computed by minimizing its TC functional^57^. Then, the argument of the refocused complex wavefront is extracted, residual optical aberrations are removed by subtracting a reference hologram without cells^58^, and the resulting phase map is denoised through the 2D windowed Fourier transform filtering^59^ and unwrapped through the PUMA algorithm^60^. At the end of this process, a 201 × 201 QPM is selected around the cell’s weighted centroid^37^, as reported in Fig. 5c,d. Therefore, each 384 × 384 holographic ROI is converted into the corresponding 201 × 201 QPM. To reconstruct the 3D RI tomogram of a single cell, the hundreds of QPMs recorded during its roto-translation along the microfluidic channel are combined together through the Filtered Back Projection (FBP) algorithm^29^, which takes in input also the corresponding viewing angles. In the TPI-FC system, the viewing angles correspond to the rolling angles of the cell, which are estimated by exploiting the microfluidic properties and the holographic tracking^55^, since cells mainly rotate around the $x$-axis with a continuous and quasi-uniform roto-translational speed.

In a conventional fluorescence imaging flow cytometry system, usually just one 2D intensity map per cell is recorded. Instead, in our TPI-FC system, hundreds of digital holograms per cell are acquired, which are used to reconstruct its 3D RI tomogram after computing the corresponding 2D QPMs. By using an interpreted programming language (i.e., Matlab^®^ 2022b) over an Intel^®^ Core™ i9-9900K CPU with a 64Gb RAM, the numerical processing to compute a QPM from the holographic ROI takes 7.71 s per frame. This numerical processing must be replicated for all the hundreds of recorded holographic ROIs related to each single cell, thus representing at this stage the actual bottleneck for reconstructing very large datasets of 3D RI tomograms (in fact, the final tomographic reconstruction based on the FBP algorithm takes just few seconds per cell). However, the holographic processing can be speeded up by 45 times through deep learning, thus reducing the computational time of the phase retrieval from 7.71 s to 0.17 s per frame, but at the cost of losing the finest details inside the QPMs^40^. Moreover, another way to reduce the computational burden is to consider that reconstruction of all the QPMs of one single cell, used to obtain its 3D RI tomogram, is a highly parallelizable process. Thus, multicore and/or multimachine implementations of the reconstruction software could run in parallel on GPUs. Instead, the computational time for detecting the holographic ROIs belonging to one single cell is much lower with respect to the computational time for their QPMs retrieval. In particular, the detection of all the cells inside each frame of the holographic sequence (see Fig. 5b) takes 0.16 s. The following process for grouping all the holographic ROIs belonging to the same cell takes about 20 s per cell. For example, for a cell running the entire FoV in 100 frames, the first detection step takes 100 × 0.16 s = 16 s, which must be added to the 20 s of the second detection step. However, it is important to specify that, while the second step is performed separately for each cell, the first step is shared by all the cells flowing along the channel at the same moment, as shown in Fig. 5b, thus it is implemented just once for all of them.

## Supplementary Information

Supplementary Information 1.

Supplementary Video 1.

## Data Availability

The datasets used and/or analysed during the current study available from the corresponding author on reasonable request.
