# Supplementary material for: Label-free liquid biopsy through the identification of tumor cells by machine learning-powered tomographic phase imaging flow cytometry
Source: Sci Rep. 2023 Apr 13;13:6042. doi: 10.1038/s41598-023-32110-9 (PMC10101968; doi:10.1038/s41598-023-32110-9)
Supplement: Supplementary file 1 — Supplementary Information 1. [file 41598_2023_32110_MOESM1_ESM.docx]

Supplementary Information of

Label-free liquid biopsy through the identification of tumor cells by machine learning-powered tomographic phase imaging flow cytometry

# Daniele Pirone1, Annalaura Montella^2,3^, Daniele G. Sirico^1^, Martina Mugnano^4^, Massimiliano M. Villone^4^, Vittorio Bianco^1^, Lisa Miccio^1^, Anna Maria Porcelli^5,6,7^, Ivana Kurelac^7,8^, Mario Capasso^2,3^, Achille Iolascon^2,3^, Pier Luca Maffettone^4^, Pasquale Memmolo^1,*^, Pietro Ferraro^1,*^

1 CNR-ISASI, Institute of Applied Sciences and Intelligent Systems “E. Caianiello”, Via Campi Flegrei 34, 80078 Pozzuoli, Napoli, Italy.

2 CEINGE Biotecnologie Avanzate, CEINGE Biotecnologie Avanzate, Napoli, Italy.

3 DMMBM, Department of Molecular Medicine and Medical Biotechnology, University of Naples “Federico II”, Napoli, Italy.

^4^ DICMaPI, Department of Chemical, Materials and Production Engineering, University of Naples “Federico II”, Piazzale Tecchio 80, 80125 Napoli, Italy.

^5^ Department of Pharmacy and Biotechnology (FABIT), University of Bologna, Bologna, Italy.

^6^ Interdepartmental Centre for Industrial Research 'Scienze della Vita e Tecnologie per la Salute', University of Bologna, Bologna, Italy.

^7^ Centre for Applied Biomedical Research (CRBA), University of Bologna, Bologna, Italy.

^8^ DIMEC, Department of Medical and Surgical Sciences, Centro di Studio e Ricerca sulle Neoplasie (CSR) Ginecologiche, Alma Mater Studiorum-University of Bologna, 40138 Bologna, Italy.

*[corre](mailto:corresponding.author@email.example)sponding authors: [pasquale.memmolo@isasi.cnr.it](mailto:pasquale.memmolo@isasi.cnr.it), [pietro.ferraro@cnr.it](mailto:pietro.ferraro@cnr.it)

|  | **Monocyte** | **Tumor cells (Types & Numbers)** | | | | | | | |
| --- | --- | --- | --- | --- | --- | --- | --- | --- | --- |
|  |  | **Neuroblastoma Cancer** | | | | | **Ovarian Cancer** | | |
|  | **THP1** | **CHP212** | | **SKNBE2** | | **SKNSH** | **A2780** | | **CAOV3** |
| **Total** | 247 | 115 | | 106 | | 151 | 95 | | 215 |
|  |  | 372 | | | | | 310 | | |
| **Training Set** | 200 | 83 | | 83 | | 84 | 80 | | 170 |
|  |  | 250 | | | | | 250 | | |
| **Augmented Training Set** | 2000 | 332 | 332 | | 336 | | 320 | 680 | |
|  |  | 1000 | | | | | 1000 | | |
| **Test Set** | 47 | 32 | | 23 | | 67 | 15 | | 45 |
|  |  | 122 | | | | | 60 | | |

**Table S1.** Summary of the tomographic dataset.

**Mathematical definition of classification metrics**

In order to quantify the performance of the proposed classifiers, several metrics have been employed. Let $TP$ be the number of true positives, $TN$ the number of true negatives, $FP$ the number of false positives, and $FN$ the number of false negatives.

The Accuracy is defined as

$ACC=\frac{TP+TN}{TP+TN+FP+FN}$. (S1)

The True Positive Rate (Sensitivity or Recall) is defined as

$TPR=SENS=REC=\frac{\mathrm{TP}}{TP+FN}$. (S2)

The True Negative Rate (Specificity) is defined as

$TNR=SPEC=\frac{\mathrm{TN}}{TN+FP}$. (S3)

The Positive Predictive Value (Precision) is defined as

$PPV=PREC=\frac{\mathrm{TP}}{TP+FP}$. (S4)

The Negative Predictive Value is defined as

$NPV=\frac{\mathrm{TN}}{TN+FN}$. (S5)

The Balanced Accuracy is defined as

$BA=\frac{TPR+TNR}{2}$. (S6)

The F1 Score is defined as

$F1=\frac{2TP}{2TP+FP+FN}$. (S7)

The Matthews Correlation Coefficient is defined as

$MCC=\frac{TP\times TN-FP\times FN}{\sqrt{\left( TP+FP \right)\left( TP+FN \right)\left( TN+FP \right)\left( TN+FN \right)}}$. (S8)

The Fowlkes–Mallows Index is defined as

$FM=\sqrt{PPV\times TPR}$. (S9)

**Supplementary Movie S1**

QPM sequence of a monocyte (THP1), a neuroblastoma cancer cell (SKNSH), and an ovarian cancer cell (CAOV3), recorded in flow cytometry modality, along with the corresponding rolling angles (first part), and slice-by-slice visualization (second part) and isolevels representation (third part) of their 3D RI reconstructions.
